# Synergy of radiotherapy, focused ultrasound, and immunotherapy in the treatment of brain metastases

**DOI:** 10.1007/s11060-025-05379-1

**Published:** 2025-12-15

**Authors:** Lucía Boffelli, Cristina Fimiani, Nicolás Gonzalo Núñez, Jenny Christine Kienzler

**Affiliations:** 1 Facultad de Ciencias Químicas, Departamento de Bioquímica Clínica, Universidad Nacional de Córdoba, Córdoba, Argentina; 2 Centro de Investigaciones en Bioquímica Clínica e Inmunología (CIBICI), Consejo Nacional de Investigaciones Científicas y Técnicas (CONICET), Córdoba, Argentina; 3 Present Address: Translational Neuroimmunology Lab, Institute of Experimental Immunology, University of Zurich, Zurich, Switzerland; 4 Present Address: Department of Neurosurgery, Lausanne University Hospital, Lausanne, Switzerland

**Keywords:** Brain metastases, Immunotherapy, Radiotherapy, Stereotactic radiosurgery, Focused ultrasound, Low-intensity focused ultrasound

## Abstract

**Purpose:**

Brain metastases (BrM) represent the most common intracranial malignancies and remain a major clinical challenge. Unlike glioblastoma (GBM), where immunotherapy has shown limited benefit, there are promising results for BrM. Nonetheless, several key aspects remain to be solved to amplify the success of these therapies, highlighting the potential of integrating immunotherapy with local strategies. This review focuses on therapeutic approaches for BrM, emphasizing the role of radiotherapy (RT) and focused ultrasound (FUS) in enhancing immunotherapy efficacy.

**Methods:**

We performed a narrative review of recent clinical studies addressing the interactions between the immune system, RT, and blood–brain barrier (BBB) modulation by FUS, with an emphasis on therapeutic strategies tested in BrM.

**Results:**

The success of immunotherapy in brain malignancies is hindered by the immunosuppressive tumor microenvironment (TME) and limited BBB penetration, as these treatments are administered systemically. RT synergizes with immunotherapy by promoting tumor antigen release and immune priming, which helps transiently overcome the immunosuppressive TME. However, excessive and prolonged antigen exposure may lead to T-cell exhaustion and checkpoint upregulation, which explains why sequential administration of stereotactic radiosurgery (SRS) followed by immunotherapy within a 2–4-week window enhances antitumor responses. Regarding the general difficulty for systemic drugs to access the brain, FUS emerges as a potent candidate for enabling transient BBB disruption, facilitating drug delivery, and biomarker access.

**Conclusion:**

Combining immunotherapy with SRS or FUS-mediated BBB modulation offers a promising path for improving outcomes in BrM. Future work must optimize these multimodal strategies while minimizing toxicity.

## Introduction

The management of primary brain tumors, such as glioblastoma (GBM), and brain metastases (BrM) presents significant challenges due to the brain’s intricate microenvironment and the restrictive properties of the blood-brain barrier (BBB) [1]. BBB is a specialized, semi-permeable structure composed of endothelial cells, astrocytic end-feet, and pericytes and it is essential for maintaining brain homeostasis and proper neuronal function [2]. Tight junctions between endothelial cells restrict paracellular diffusion, allowing mainly small, lipophilic molecules to traverse by passive diffusion. Although this barrier confers critical neuroprotection, it simultaneously impedes the effective delivery of many therapeutic agents to the central nervous system (CNS), thereby limiting drug efficacy and contributing to the failure of several treatments for brain disorders [2].

**Fig. 1:**
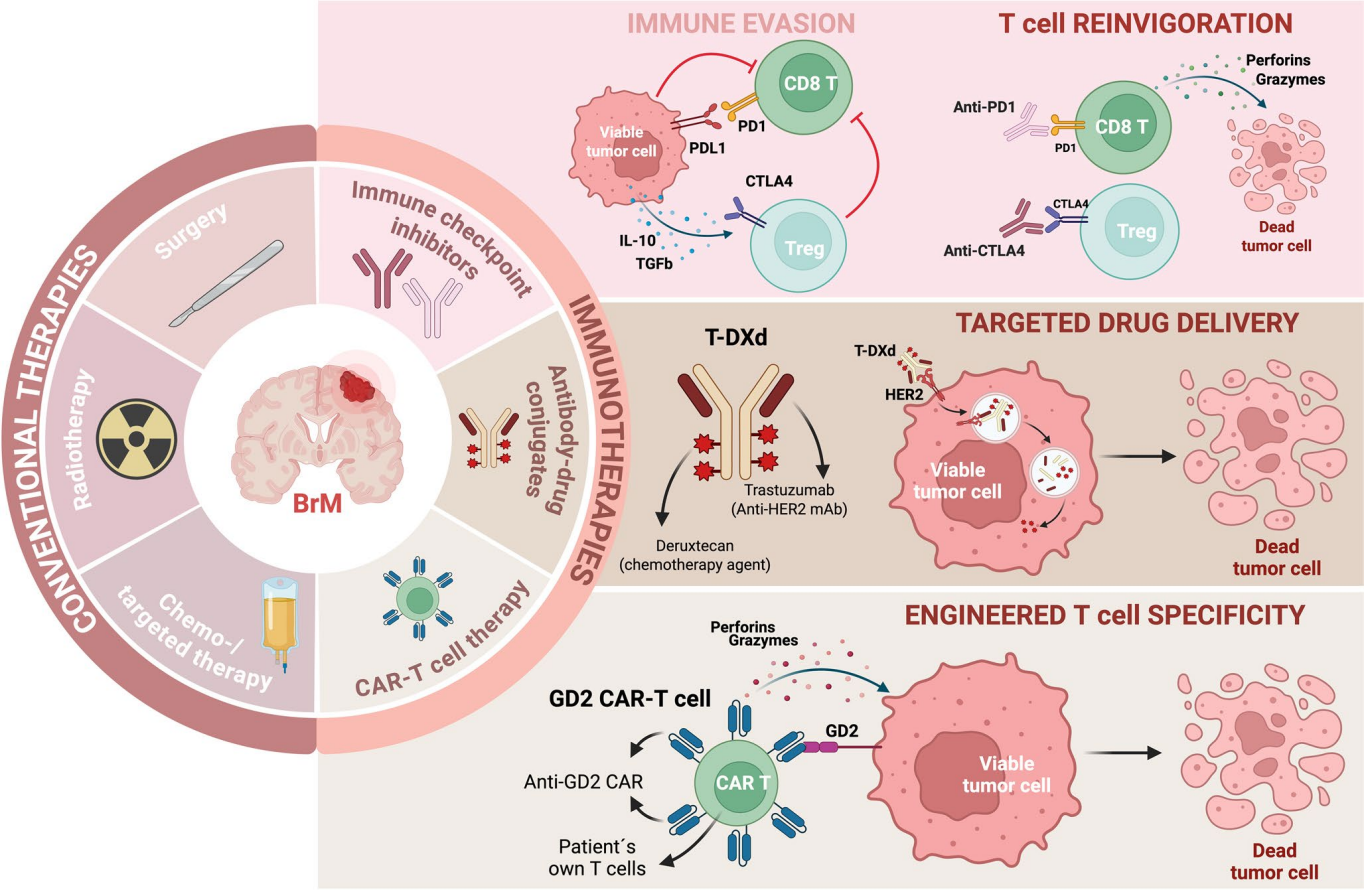
Conventional therapies vs. Immunotherapies for BrM treatment. On the left panel, conventional therapies for the treatment of these tumors include local therapies like surgery and RT, and systemic therapies such as chemotherapy and targeted therapy. On the right panel, emerging immunotherapies are depicted in the field of BrM, like ICIs (resumed mechanism on the top right panel), ADC (mechanism explained with T-DXd example, middle right panel) and CAR-T cell therapy (resumed mechanism on the bottom right panel). Figure made with Biorender

Current standard-of-care therapies for BrM include surgery, radiotherapy (RT), chemotherapy, and targeted therapies [3–5] (Fig. 1). In current clinical practice, surgical resection is typically reserved for selected patients, particularly when a metastasis produces substantial mass effect, causes significant peritumoral edema, or when tissue is required for diagnostic clarification. Surgery is most often considered in cases of single or oligometastatic disease with surgically accessible lesions, radio-resistant histologies such as melanoma or renal cell carcinoma, posterior fossa tumors at risk of precipitating obstructive hydrocephalus, or suspected radionecrosis or progression following prior radiation [6]. It is also favored in patients with good functional status and controlled systemic disease [7, 8]. In contrast, asymptomatic lesions without mass effect are generally treated with RT, depending on lesion number, size, and distribution.

RT is delivered either as whole-brain radiotherapy (WBRT) or as single-dose or fractionated stereotactic radiosurgery (SRS), a high dose of precisely targeted radiation [9]. Historically, WBRT was the predominant RT modality for patients with BrM. However, growing concerns about neurocognitive toxicity and advances in focal radiation techniques have driven a progressive shift toward SRS [10]. In parallel, there has been a rise in the use of systemic therapies in combination with radiation (from 26.4% to 36.5%), illustrating the contemporary shift toward increasingly multimodal treatment paradigms [11].

Traditionally, SRS was reserved for patients with a limited number of lesions [12], but currently there is no universal consensus on the maximum number, size, or location of metastases appropriate for SRS. Evidence from the Japanese Gamma Knife trial and more recent fractionated SRS series supports the use of SRS in patients with up to 10–15 lesions [13–15], and emerging reports describe favorable outcomes in carefully selected patients with even >20 metastases [16, 17]. WBRT remains an option for patients with diffuse intracranial disease, while SRS to the surgical cavity is frequently used after resection to improve local control. By contrast, systemic therapies such as chemotherapy and targeted agents have historically shown limited benefit due to the BBB and variable drug penetration; however, advances in tyrosine kinase inhibitors (TKIs) and antibody–drug conjugates (ADCs) are beginning to shift this paradigm [18] (Fig. 1). Importantly, tumor histology also influences treatment selection, as certain histologies respond more favorably to focal SRS compared with WBRT, further underscoring the need for individualized therapeutic decision-making [19].

For GBM, the standard regimen is maximal safe surgical resection followed by concurrent RT with the chemotherapy agent temozolomide and subsequent adjuvant temozolomide [20]. Despite this multimodal approach, prognosis remains poor, with median overall survival (OS) of approximately 15–18 months, underscoring the urgent need for more effective systemic therapies.

## Immunotherapy in BrM

Immunotherapies have emerged as a therapeutic alternative that aims to harness and enhance the patient’s own immune system to recognize and eliminate tumor cells. In the context of brain tumors, this approach requires a detailed understanding of the immune cell composition within the tumor microenvironment (TME) to identify opportunities and constraints for effective immune modulation.

### Immune landscape of BrM vs. GBM

While both BrM and GBM arise within the central nervous system, they differ substantially in their cellular origin, immunologic profile, and therapeutic responsiveness. BrM typically retain key immunologic features of their extracranial primaries and often display higher lymphocytic infiltration [21, 22], whereas GBM is marked by profound immunosuppression, myeloid predominance, and abundant tumor-associated macrophages [23–25]. These biological differences have important therapeutic implications: BrM, particularly melanoma or lung cancer metastases, generally respond better to immune checkpoint inhibitors (ICI), while GBM remains largely resistant [26, 27]. Understanding these distinctions is essential when evaluating synergistic strategies combining RT [28–30] or focused ultrasound (FUS) [31–33] with immunotherapy, as mechanisms of immune activation and treatment sensitivity differ markedly between metastatic and primary tumors. Importantly, recognizing these divergent immune landscapes is also critical for tailoring and developing new immunotherapeutic approaches specifically suited to each tumor type.

Although the brain has long been considered an immune-privileged site, it is now well established that it can mount effective immunogenic responses, including those directed against tumors [34]. In the healthy brain, immunosurveillance is primarily carried out by microglia, the resident macrophages of the CNS. Additional immune populations, including dendritic cells (DCs), T cells, border-associated macrophages, and monocyte-derived macrophages, also contribute to tissue integrity and immune homeostasis [35].

This delicate balance is profoundly altered in the presence of tumors. Primary brain tumors such as GBM exhibit pervasive myeloid dominance, effector T cell exclusion, accumulation of regulatory T cells (Tregs), and potent local immunosuppression, contributing to repeated failures of immunotherapies in clinical trials [36]. In contrast, BrM arise from peripheral tumors that colonize the CNS, and thus their immune landscapes reflect both the biology of the primary tumor and the unique constraints of the brain microenvironment. This dual influence generates distinct patterns of immune cell infiltration and activation, with key implications for immunotherapy responsiveness [37]. BrM, especially those originating from melanoma or certain lung cancers, often contain higher densities of tumor-infiltrating lymphocytes (TILs) and exhibit more immunoreactive states.

However, despite their comparatively higher immunogenicity, BrM still develop a markedly immunosuppressive TME [38–40] and remain partly shielded by the BBB, limiting the penetration and efficacy of therapies such as immune checkpoint inhibitors (ICI) [41]. Thus, even in BrM, improving immune cell trafficking and therapeutic delivery across the BBB remains a critical challenge and a central motivation for strategies such as focused ultrasound or radiation-induced BBB modulation.

Spatial transcriptomic and single-cell studies further highlight these distinctions: BrM samples show heterogeneous immune niches encompassing both exhausted and active T cell populations, whereas GBM presents comparatively uniform, myeloid-driven immunosuppression and fewer cancer-reactive T cells [42].

Recent work has highlighted the importance of the glymphatic system, a perivascular network involved in cerebrospinal fluid circulation and metabolic waste clearance. This system contributes to antigen drainage from the CNS and plays an important role in immune surveillance. Studies by Kipnis and colleagues have demonstrated that glymphatic flow supports communication between CNS tissues and peripheral immune organs, adding an additional layer of complexity to neuroimmune interactions [43].

### Immune checkpoint inhibitors (ICI)

Immune checkpoint molecules are inhibitory pathways that regulate T cell activation and maintain peripheral tolerance, thereby preventing autoimmunity and excessive tissue damage. Among the best-characterized targets are cytotoxic T-lymphocyte antigen 4 (CTLA-4), which dampens early T cell priming, and programmed cell death protein 1 (PD-1), which inhibits T cell effector function within tissues [44]. Blocking these pathways with ICI can reinvigorate antitumoral T cell responses and has transformed the treatment landscape of several solid tumors. (Fig. 1).

It is in this context that monoclonal antibodies (mAb) targeting immune checkpoint receptors were developed and approved for the treatment of several metastatic cancers [45, 46]. In melanoma, multiple ICI, including nivolumab and pembrolizumab (anti-PD1 mAb) and ipilimumab (anti-CTLA-4 mAb), are FDA-approved for unresectable or metastatic disease, either as monotherapy or in combination (nivolumab plus ipilimumab) [47]. Importantly, these approvals include patients with BrM, supported by clinical trials demonstrating durable intracranial responses and prolonged survival [48–52]. In non-small cell lung cancer (NSCLC), several ICI are also approved for metastatic disease, such as pembrolizumab, nivolumab, and cemiplimab (anti-PD1 mAb) and atezolizumab and durvalumab (anti-PDL1 mAb), either as monotherapy or in combination with chemotherapy, depending on PDL1 expression and treatment setting [53–55]. These agents have shown intracranial efficacy, although response rates are generally lower than those observed in melanoma. In breast cancer, ICI approval is currently limited to the triple-negative subtype (TNBC) [56, 57]. Pembrolizumab, in combination with chemotherapy, is FDA-approved for patients with PDL1–positive (CPS ≥ 10) unresectable locally advanced or metastatic TNBC [58]. However, single-agent activity and efficacy in brain metastases remain limited.

### Targeted therapies and ADC with CNS activity

Antibody–drug conjugates (ADCs) represent an emerging immunotherapeutic modality for tumors that frequently metastasize to the brain [58, 59], such as HER2 + breast cancer (Fig. 1) [60, 61]. In breast cancer, HER2-directed ADCs, such as trastuzumab emtansine (T-DM1) and trastuzumab deruxtecan (T-DXd), have been practice-changing, with T-DXd producing durable responses and activity even in HER2-low disease [62, 63]. Importantly, these studies include patients with metastatic disease, and emerging data show intracranial responses in HER2-positive BrM. T-DXd has also been approved for HER2-mutant NSCLC, with early signals of CNS efficacy in metastatic cases [64]. Likewise, the Trop-2–directed ADC sacituzumab govitecan has demonstrated benefit in metastatic breast cancer, including patients with brain metastases [65].

However, their penetration across the intact BBB remains limited [66]. This challenge has increased interest in focused ultrasound (FUS)-mediated BBB opening, which has been shown in preclinical models to enhance ADC delivery to intracranial lesions [67, 68]. Therefore, while ADCs are not yet widely studied in combination with RT or FUS, their dependence on BBB permeability makes them a conceptually relevant platform for future synergistic approaches.

### Chimeric antigen receptor (CAR)T cells therapy in BrM

CAR-T cell therapies have demonstrated remarkable efficacy in hematologic malignancies [69], but their application to CNS tumors, including GBM and BrM, remains constrained by limited trafficking across the BBB, antigen heterogeneity, and a highly immunosuppressive microenvironment [70, 71]. Despite these challenges, early proof-of-concept studies have shown that CAR-T cells can be delivered safely to the CNS and induce antitumor responses, particularly when administered intraventricularly or intratumorally, thereby partially bypassing the BBB. Examples include GD2-directed CAR-T cells (Fig. 1) in diffuse midline gliomas (NCT03170141) [72] and additional early-phase trials targeting B7-H3 (NCT04185038) [73], EGFRvIII (NCT01454596) [74], and HER2 (NCT01109095) [75]. While most ongoing studies focus on GBM and pediatric midline gliomas, clinical investigations in BrM remain scarce; among the few, HER2-CAR-T therapy for metastatic breast cancer with brain involvement (NCT03696030) exemplifies the potential for target-defined applications in selected metastases.

These biological and anatomical barriers have prompted increasing interest in combining CAR-T therapy with BBB-modulating approaches. Strategies such as focused ultrasound or radiation have been shown in preclinical intracranial models to enhance CAR-T trafficking and intratumoral accumulation [76, 77]. Although CAR-T is not yet a clinically established modality for BrM, it illustrates how transient BBB disruption, including FUS or RT-induced modulation, may unlock new opportunities for cellular immunotherapies within the CNS.

## Combination of RT and immunotherapy for BrM

**Fig. 2:**
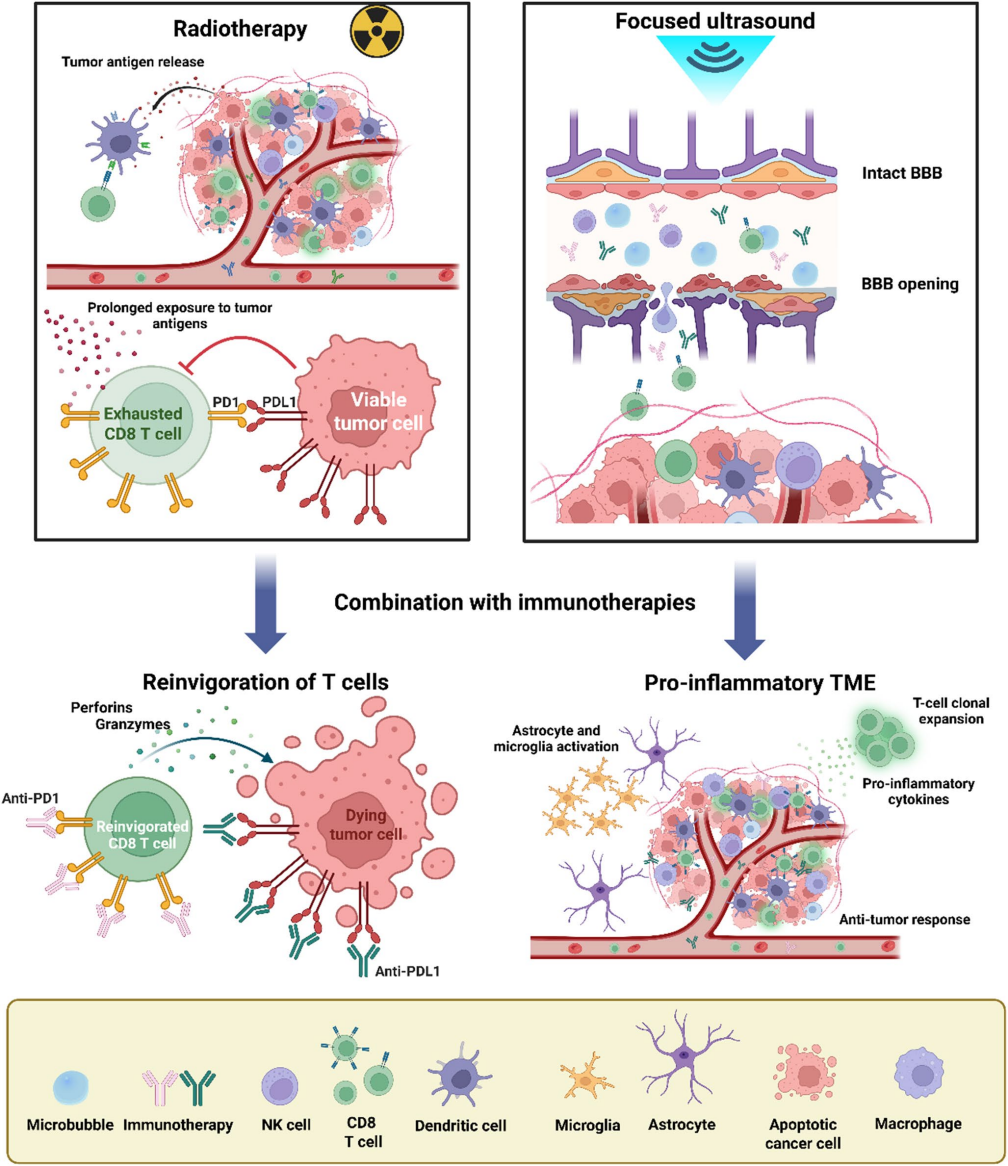
Mechanisms by which RT and FUS may enhance immunotherapy efficacy. Application of RT induces tumor antigen release, potentially facilitating tumor recognition and enabling DCs to capture and present antigens to CD8⁺ T cells. Nevertheless, the constant exposure to tumor antigens can cause T cell exhaustion, and overexpression of checkpoint molecules such as PD1/PDL1. Administration of ICIs after RT can partially solve this issue and reinvigorate exhausted T cells. On the other hand, FUS alters the BBB, increasing the likelihood of immune cells and drugs penetrating the CNS. Together, these approaches may help overcome key challenges that immunotherapies face in accessing the CNS and counteracting tumor-induced immunosuppression in the brain. Figure made with Biorender

The convergence of RT and ICI has generated considerable interest, as there is evidence that the concurrent use of these treatment modalities might improve their efficacy. The mechanism, whereby radiation converts tumors into in situ vaccines through antigen release and inflammatory signaling, provides the biological rationale for combination therapy. There are different mechanisms that favor the positive effect of radiation on immune activation, such as: the increase in density of TILs and particularly cytotoxic T cells, the activation of DCs, and the overexpression of immune checkpoint molecules [78] (Fig. 2).

### Clinical efficacy

The combination of RT with immunotherapy demonstrates a generally positive effect on survival outcomes. In a propensity-matched study, the treatment effect of RT with ICI was compared to RT and chemotherapy in a NSCLC cohort. They found that patients receiving RT with immunotherapy after neurosurgical resection achieved a median overall survival of 23 months compared to 11.8 months with RT and chemotherapy [79]. In a recent study in patients with NSCLC, the intracranial overall response rate was 49.1% in the ICI-treated patients compared to 75.9% in the ICI and RT co-treated patients [80]. Similarly, in another cohort of NSCLC patients, OS was significantly longer in a group that received ICI with upfront RT compared to ICI alone, whereas this benefit was not present in patients who received ICI and chemotherapy [81]. Consistent with these findings, a study on melanoma BrM patients treated with RT and ICI showed improved outcomes when RT was administered before ICI, with higher overall response rate (HR 7.88) and disease control rate (HR 6.26) [82]. Another study in melanoma patients after BrM resection revealed that administering RT before ICI was superior to giving ICI prior to RT [83]. Overall, these studies consistently support the benefit of combining RT with ICI over RT with chemotherapy or ICI alone. Importantly, sequencing appears critical, with superior outcomes when RT precedes ICI rather than the reverse.

A more in-depth focus is needed on SRS, as it drives a stronger pro-inflammatory response and in situ immunization compared to conventional RT [84, 85]. Clinical evidence supports the benefit of combining SRS with ICI, particularly when treatments are delivered within a short time window. For instance, in a cohort of BrM patients, administration of SRS in close temporal proximity to ICI (within four months) resulted in a one-year local control rate of 90.3% [86]. However, evidence indicates that even narrower intervals may provide superior benefit. In a retrospective study of 580 BrM, concurrent SRS-ICI (ICI within 4 weeks of SRS) was associated with lower rates of extracranial failure and improved OS compared to non-concurrent therapy [87]. Consistently, another study demonstrated that concurrent application of SRS within four weeks of ICI yielded greater lesion volume reduction at 1.5, 3, and 6 months compared to non-concurrent schedules [88].

Other studies have highlighted additional nuances. For instance, Le et al. reported that in NSCLC and melanoma BrM, concurrent SRS-ICI (< 30 days) did not improve local control but significantly reduced distant brain failure compared to non-concurrent schedules [89]. In a phase II trial including 26 patients, combining ICI and SRS within 14 days was well tolerated and prolonged 1-year PFS to 45.2% [90]. Similarly, Kotecha et al. compared lesions treated with concurrent ICI (± 5 half-lives) to lesions treated with delayed ICI, finding superior overall response and durability, particularly in patients receiving immediate ICI (± 1 half-life; 12-month durable response 94% vs. 71%). Notably, lesions pre-exposed to ICI responded less favorably than ICI-naïve lesions [91]. Finally, in another study, concurrent SRS (defined as ICI given within two weeks of RT) was associated with improved OS compared to sequential strategies and predicted a reduced risk of developing ≥ 3 new BrM after SRS [92].

The most consistent finding across studies involves intracranial disease control. Perhaps most intriguingly, several investigations found reduced rates of new brain metastases development, suggesting the combination may prevent distant brain failure beyond treating existing lesions.

### Treatment sequencing and timing

The optimal sequencing appears to favor RT before immunotherapy. The proposed mechanism suggests radiation first primes the immune system through antigen presentation, which subsequent ICI then amplifies. Conversely, administering immunotherapy first may result in immune activation being subsequently suppressed by radiation-induced inflammatory changes. Timing emerges as another critical variable. Most studies define concurrent therapy as administration within two to four weeks, with several demonstrating superior outcomes when treatments occur earlier within this window.

### Safety considerations

The safety profile of combination therapy is generally safe, with a mildly increased risk for radiation necrosis (RN) in patients treated with ICI and SRS compared to SRS alone. Radiation necrosis rates vary considerably across studies, ranging from 1.5% to 14% [82, 90–93]. Most RN remains asymptomatic, detected only on imaging. Tumor volume consistently emerges as the strongest predictor of toxicity rather than treatment timing or sequencing. Cabanie et al.’s analysis confirmed volume as the only significant predictive factor for radiation necrosis [93]. Patient selection therefore becomes crucial, with larger tumors potentially warranting modified approaches or closer monitoring. The heterogeneity in reported toxicity rates likely reflects differences in patient populations, tumor characteristics, and treatment protocols. Studies including patients with larger tumors, melanoma histology, or requiring corticosteroids report higher complication rates. This variation underscores the importance of individualizing treatment decisions based on specific clinical factors.

### Clinical implications

Current evidence suggests that combining SRS with immunotherapy offers meaningful benefits for selected patients with BrMs, particularly regarding intracranial disease control. The optimal approach likely involves administering RT before or concurrently with immunotherapy within a two – four-week window.

The acceptable safety profile supports clinical implementation, though careful patient selection remains essential. Patients with smaller tumor volumes and those not requiring corticosteroids appear best suited for combination therapy. Regular monitoring for radiation necrosis is warranted, particularly in higher-risk populations.

While these findings are encouraging, the evidence base remains largely retrospective with inherent selection biases. Ongoing prospective trials will be crucial for definitively establishing optimal protocols and identifying patients most likely to benefit from this promising therapeutic combination.

## Combination of focused ultrasound and immunotherapy for BrM

Focused ultrasound (FUS) represents a promising, non-invasive tool that enables transient and localized BBB disruption, thereby facilitating targeted drug delivery to neoplastic tissue or, alternatively, inducing direct ablation of tumor structures without reliance on surgical intervention or ionizing radiation [32] (Fig. 2).

### Mechanisms of action and applications

FUS employs highly focused acoustic waves to concentrate energy within a defined intracranial region. Depending on the applied acoustic parameters, such as frequency and intensity, FUS can elicit a range of therapeutic responses and can be divided into high-intensity focused ultrasound (HIFU) and low-intensity focused ultrasound (LIFU). HIFU is generally used for tissue ablation, whereas LIFU is often used for BBB opening and microenvironmental regulation (facilitating neuromodulation and drug delivery) [94, 95].

### Tumor ablation

Tissue ablation by HIFU can be categorized into thermal and mechanical ablation. Thermoablation occurs when ultrasound waves are converted to heat in biological tissue, raising the temperature and resulting in tumor cell necrosis or coagulative destruction [94]. Mechanical ablation (histotripsy) is related to the cavitation effect, resulting in mechanical tissue destruction and fragmentation [96]. Unlike SRS or WBRT, which use ionizing radiation, HIFU is non-ionizing and highly localized, sparing healthy tissue and avoiding cumulative radiation toxicity. It may be preferred in patients not eligible for further radiotherapy due to prior dose limits. However, its clinical use is still limited compared with SRS/WBRT, and ongoing studies are defining its precise role.

### Enhanced delivery of immunotherapy

BBB opening is primarily achieved when LIFU is used in combination with intravenously administered microbubbles (MB). The inertial cavitation of these MB following ultrasound delivery generates intravascular shear stress, which causes BBB endothelial cell tight junctions to become loose and open reversibly for a short time [97]. This mechanism can promote the entry of therapeutic compounds into brain tumors and increase their concentrations within tumor tissues. The first preclinical study to demonstrate this concept documented the delivery of trastuzumab into a mouse brain via BBB opening induced by FUS and MB (FUS-MB) [98]. In a breast cancer BrM model, the median survival was increased by 32% through the combined use of trastuzumab and FUS versus trastuzumab treatment alone [99]. Subsequently, the impact of FUS-mediated BBB disruption on the transport of two anticancer agents was examined (doxorubicin and T-DM1) in an orthotopic xenograft model of HER2-positive breast cancer BrM, demonstrating increased uptake and penetration of these compounds [100]. Translating these findings to clinical application, magnetic resonance-guided focused ultrasound (MRgFUS), has enabled safe, non-invasive delivery of trastuzumab in patients with HER2-positive BrM [68]. Trastuzumab uptake in sonicated BrM was increased by 101% on average, which correlated with a decrease in tumor size of 19% [68]. Building on these promising results, ongoing clinical trials examine the safety and efficacy of the FUS-assisted delivery of ICI, including nivolumab (NCT04021420) and pembrolizumab (NCT05317858) into the CNS for the treatment of metastatic melanoma and metastatic NSCLC [101].

### Modulation of tumor immune microenvironment

LIFU-mediated opening of the BBB has the capacity to modulate the tumor immune microenvironment in the brain through both mechanical and thermal mechanisms. Following LIFU treatment, there is increased expression of pro-inflammatory cytokines, chemokines, and adhesion molecules [33], followed by the infiltration of neutrophils into the brain parenchyma [102] and the activation of astrocytes and microglia [103]. This intervention has the potential to reprogram “cold” tumors into “hot” tumors, which may foster sustained anti-tumor immune responses [104]. The combination of LIFU with ICIs has been shown to enhance responses to immunotherapy in preclinical glioma models [95]. Specifically, anti-PD-1 antibodies delivered through the opened BBB can block immune exhaustion of effector T cells, thereby enabling T cells to exert effector responses through perforin and/or granzyme B [95]. Additionally, it has been demonstrated that LIFU-facilitated delivery of CAR-T cells allows these cells to infiltrate the TME more diffusely and with greater persistence, thereby enhancing tumor cytotoxicity through the CARs [95].

### Sonobiopsy

Sonobiopsy is a novel, minimally invasive technique that uses FUS combined with MB to temporarily open the BBB and release tumor-derived biomarkers into the bloodstream for non-invasive molecular diagnosis of brain tumors [105]. By targeting specific tumor regions, sonobiopsy enables spatially selective enrichment of circulating tumor DNA and other molecular biomarkers, significantly improving detection sensitivity compared to conventional plasma-based liquid biopsies [106]. Therefore, sonobiopsy represents a promising advancement toward precision medicine in neuro-oncology, enabling more accurate tumor characterization and monitoring while avoiding risks associated with invasive tissue sampling procedures.

## Concluding remarks

The treatment of CNS tumors, whether primary or secondary, remains hampered by the restrictive BBB and the immunosuppressive TME. Combining local therapies with immunotherapy has emerged as a promising way to overcome these barriers. RT, and especially SRS, is already broadly implemented in clinical practice and can act as an in situ vaccine by promoting antigen release and immune priming, although optimal timing with ICI is critical to avoid T-cell suppression and exhaustion and to maximize therapeutic efficacy. In contrast, FUS-mediated BBB opening is being evaluated in early clinical trials and has yet to transition into standard clinical practice. While FUS enables transient BBB disruption to facilitate the delivery of drugs and immune cells into the brain, alongside direct antitumor and immune-modulating effects, its clinical application is still investigational. Translating FUS approaches into routine care will require overcoming several regulatory and technical barriers, including the standardization of BBB-opening parameters, characterization of BBB reclosure kinetics, reproducibility across centers, and the establishment of long-term safety profiles. Moreover, significant knowledge gaps remain regarding the optimal timing of RT or FUS relative to immunotherapy, as well as the precise mechanisms through which FUS may enhance the trafficking or efficacy of immune-based therapies across the BBB.

As these challenges are addressed, multimodal approaches integrating RT and FUS with immunotherapy hold substantial promise for improving intracranial tumor control and survival. Nonetheless, uncertainties persist regarding the safety of BBB disruption, the durability of the induced immune responses, and the identification of ideal sequencing regimens. Future prospective studies will be essential to refine these strategies, delineate their clinical readiness, and fully harness their synergistic potential (Table 1).

**Table 1:** Clinical trials terminated or ongoing for the treatment of BrM with immunotherapy alone or in combination with RT or FUS

ID	Title	Phase	Treatment	# patients	Study design	Primary Histology	Timing	Outcome
Immunotherapies								
NCT02374242	A Phase II Study of Nivolumab and Nivolumab Combined With Ipilimumab in Patients With Melanoma Brain Metastases	II	Nivolumab, nivolumab + ipilimumab	76	Randomized, parallel assignment, open label, treatment purpose.	Melanoma	Concurrent	Intracranial response rate (primary); ORR; intracranial/extracranial/overall PFS; OS; safety (CTCAE).
NCT05012254	Nivolumab and Ipilimumab Plus Chemotherapy for Patients With Stage IV Lung Cancer With Brain Metastases	II	Chemotherapy + nivolumab + ipilimumab	71	Single-group, open-label interventional study (treatment purpose).	Lung cancer	Concurrent	Intracranial clinical benefit rate (RANO-BM). PFS (systemic & intracranial), ORR (intracranial & systemic), safety (CTCAE v5.0).
NCT02477826	An Open-Label, Randomized Phase 3 Trial of Nivolumab, or Nivolumab Plus Ipilimumab, or Nivolumab Plus Platinum Doublet Chemotherapy Versus Platinum Doublet Chemotherapy in Subjects With Chemotherapy-Naïve Stage IV or Recurrent Non-Small Cell Lung Cancer (NSCLC)	III	Chemotherapy + Nivolumab + Ipilimumab	2748	Randomized, parallel assignment, open label, treatment purpose.	NSCLC	Concurrent	PFS (BICR, RECIST 1.1), OS. ORR (BICR), symptom deterioration (LCSS).
NCT03526900	Phase II Non-randomized Study of a Atezolizumab (MPDL3280A) in Combination With Carboplatin Plus Pemetrexed in Patients Who Are Chemotherapy-naïve and Have Stage IV Non-squamous Non-small Cell Lung Cancer with Asymptomatic Brain Metastasis	II	Chemotherapy + Atezolizumab	40	Single-group, open-label interventional study (treatment purpose).	NSCLC	Concurrent	PFS rate at 12 weeks (RECIST 1.1 / RANO). ORR (RECIST / RANO), steroid-adjusted response (RANO), time to brain radiotherapy.
NCT04211090	A Phase II Study to Evaluate Camrelizumab With Pemetrexed / Carboplatin in Patients With Brain Metastases of Driven Gene-negative, Non-squamous Non-small Cell Lung Cancer	II	Chemotherapy + Camrelizumab	45	Allocation: N/A; Interventional model: Single group assignment; Population: Patients with brain metastases from driver gene–negative, non-squamous NSCLC; Masking: None (open label); Primary purpose: Treatment.	NSCLC	Concurrent	Intracranial objective response rate (iORR). Intracranial PFS (iPFS); ORR; PFS; safety (AEs, CTCAE v4.03).
NCT02085070	A Phase 2 Study of MK-3475 in Patients With Metastatic Melanoma and Non-Small Cell Lung Cancer With Untreated Brain Metastases	II	Pembrolizumab	65	Non-Randomized, single group assignment, open label, treatment purpose.	Melanoma, NSCLC	NA	Intracranial OR (mRECIST). Intracranial response assessment (mRECIST).
NCT04507217	A Phase II, Open-Label, Multicenter, Prospective Clinical Study to Investigate the Efficacy and Safety of Tislelizumab Combined With Pemetrexed/ Carboplatin in Patients With Brain Metastases of Non-squamous Non-small Cell Lung Cancer	II	Chemotherapy + Tislelizumab	36	Single-group assignment; open-label; N/A allocation; treatment purpose.	NSCLC	Concurrent	PFS rate at 12 months (RECIST 1.1). ORR; PFS (RECIST 1.1); OS; PFS (RANO-BM); DoR; safety (CTCAE v5.0); neurocognitive function (HVLT-R). PD-L1, TMB, exploratory biomarkers; intracranial PFS (RECIST 1.1 / RANO-BM).
NCT00623766	A Multi-Center Phase II Study to Evaluate Tumor Response to Ipilimumab (BMS-734016) Monotherapy in Subjects With Melanoma Brain Metastases	II	Corticosteroid + Ipilimumab	99	Non-Randomized, single group assignment, open label, treatment purpose.	Melanoma	NA	Disease Control Rate DCR (mWHO). DCR (irRC); best overall response BOR (mWHO/irRC); Duration of Response DoR; PFS; OS; safety (AEs, irAEs, SAEs); onset of response
NCT03563729	Efficacy of Immunotherapy in Melanoma Patients With Brain Metastases Treated With Steroids	II	Corticosteroid + Ipilimumab/Nivolumab or Pembrolizumab	80	Non-Randomized, Parallel Assignment, open label, treatment purpose.	Melanoma	Sequential	6-month PFS rate; 6-month OS rate. PFS; OS; ORR; extracranial response rate; intracranial response rate; intracranial clinical benefit rate; blood/tissue biomarkers.
NCT01654692	A Phase II Study of the Combination of Ipilimumab and Fotemustine in Patients With Unresectable Locally Advanced or Metastatic Malignant Melanoma	II	Chemotherapy + Ipilimumab	86	Single-group assignment; open-label; N/A allocation; treatment purpose.	Melanoma	Concurrent	irDCR. safety; irMDDCR; irORR; irTTR; irPFS; brain-PFS; OS.
NCT02320058	A Multi-Center Phase 2 Open-Label Study to Evaluate Safety and Efficacy in Subjects With Melanoma Metastatic to the Brain Treated With Nivolumab in Combination With Ipilimumab Followed by Nivolumab Monotherapy	II	Ipilimumab + Nivolumab	119	Single-group assignment; open-label; N/A allocation; treatment purpose.	Melanoma	Concurrent	Intracranial Clinical Benefit Rate (CBR). modified RECIST 1.1. CNS Clinical Benefit Rate (CBR). Intracranial ORR (CR + PR) and PFS
NCT06640582	Efficacy and Safety of Autologous Tumor-Infiltrating Lymphocytes (TIL) Therapy Combined With Pembrolizumab Immunotherapy in Patients With Advanced Brain Cancer Including Gliomas and Meningiomas	I/II	TILs + Pembrolizumab	85	Single-group assignment; open-label; N/A allocation; treatment purpose.	Brain Cancer, Glioma, Glioblastoma, Meningioma	Concurrent	Adverse Events (AEs). ORR, DCR, DOR, and PFS
NCT03470922	A Randomized, Double-Blind Phase 2/3 Study of Relatlimab Combined With Nivolumab Versus Nivolumab in Participants With Previously Untreated Metastatic or Unresectable Melanoma	II/III	Relatlimab + Nivolumab	714	Randomized, Parallel Assignment, quadruple-blinded, treatment purpose.	Melanoma	Concurrent	PFS (RECIST 1.1, BICR). OS, ORR, DOR, DCR, AEs
NCT03696030	A Phase 1 Cellular Immunotherapy Study of Intraventricularly Administered Autologous HER2-Targeted Chimeric Antigen Receptor (HER2-CAR) T Cells in Patients With Brain and/or Leptomeningeal Metastases From HER2 Positive Cancers	I	HER2-CAR T cell	24	Non-Randomized, single group assignment, open label, treatment purpose.	Breast cancer	NA	Incidence of dose-limiting toxicities (DLTs). treatment-related AEs. immune cell subsets + cytokines + circulating tumor cells (CSF & blood), CNS/systemic response (RANO/RECIST), CNS-PFS, OS, TME analyses, HER2 expression, tumor-growth modeling.
IMMUNOTHERAPY + RADIOTHERAPY								
NCT05584267	Multi-omics Evaluation System and Preferred Mode of Immune Treatment of Brain Metastasis of Lung Cancer Combined With Large-segmentation Precision Radiotherapy	II	RT + chemotherapy + immunotherapy	140	Randomized, parallel assignment, open label, treatment purpose.	NSCLC	Concurrent	Intracranial progression-free survival. sPFS, ORR, OS, AEs.
NCT05703269	Hypofractionated Radiotherapy vs. Single Fraction Radiosurgery for Brain Metastasis Patients on Immunotherapy (HYPOGRYPHE)	III	SRS + ICI (anti-PD1/anti-PDL1)	244	Randomized, Sequential Assignment, Open Label, Supportive Care purpose.	NSCLC, Renal Cell Carcinoma, Breast Carcinoma, Melanoma, SCLC	Sequential	Adverse Radiation Effect (ARE)
NCT06702826	Efficacy and Safety of Cadonilimab Combined With Stereotactic Radiotherapy as Second-line Treatment for Brain Metastases From Non-small Cell Lung Cancer (NSCLC): a Single-arm, Open-label, Phase II Clinical Trial	II	SRS + Cadonilimab (anti-PD1 anti-CTLA4 biespecific antibody)	20	Single-group assignment; open-label; N/A allocation; treatment purpose.	NSCLC	Concurrent	iORR, PFS, iPFS, OS.
NCT03340129	A Phase II, Open Label, Randomised, Controlled Trial of Ipilimumab and Nivolumab With Concurrent Intracranial Stereotactic Radiotherapy Versus Ipilimumab and Nivolumab Alone in Patients With Melanoma Brain Metastases.	II	Nivolumab + Ipilumimab + SRS	218	Randomized, parallel assignment, open label, treatment purpose.	Melanoma	Concurrent	Intracranial response rate, Extracranial response rate, ORR, OS, PFS, AEs.
NCT05522660	A Multicentre Randomised Open-label Phase III Study of Stereotactic Radiosurgery, in Addition to Standard Systemic Therapy for Patients With Metastatic Melanoma or Newly Diagnosed Metastatic NSCLC and Asymptomatic or Oligo-symptomatic Brain Metastases	III	Nivolumab + Ipilumimab + SRS	180	Randomized, parallel assignment, open label, treatment purpose.	Melanoma, NSCLC	Concurrent	CNS-specific PFS, locally assessed as per iRANO criteria
NCT02978404	A Phase II, Multi-centre Study, of Combining Radiosurgery and Nivolumab in the Treatment of Brain Metastases From Non-small Cell Lung Cancer and Renal Cell Cancer	II	SRS + Nivolumab	26	Single-group assignment; open-label; N/A allocation; treatment purpose.	NSCLC, Renal Cell Carcinoma, Melanoma, SCLC	Concurrent	OS, PFS, toxicity.
NCT06839560	A Prospective, Multicenter, Randomized Controlled Phase II Study to Evaluate the Efficacy and Safety of PD-L1 Monoclonal Antibody Combined with VEX Metronomic Chemotherapy and Concurrent or Delayed Radiotherapy in Patients with Advanced HER2-Negative Breast Cancer with Brain Metastasis	II	Chemotherapy + Avelumab + RT	102	Randomized, parallel assignment, open label, treatment purpose.	Breast cancer	Concurrent/sequential	Intracranial Progression-Free Survival (iPFS)
IMMUNOTHERAPY + FUS								
NCT03714243	A Study to Evaluate the Safety and Feasibility of Blood-Brain Barrier Disruption Using MRI-Guided Focused Ultrasound in the Treatment of Her2-positive Breast Cancer Brain Metastases	NA	FUS + Trastuzumab	10	Non-randomized, Single Group Assignment, Prospective, single arm, single center, Open Label, Device Feasibility	Breast cancer	Concurrent	Adverse events, Feasibility of BBBD.
NCT05317858	Blood-brain Barrier (BBB) Opening Using Exablate Focused Ultrasound with Standard of Care Treatment of NSCLC Brain Mets (LIMITLESS)	III	FUS + nivolumab, ipilimumab, pembrolizumab, atezolizumab, cemiplimab	30	Randomized, parallel assignment, Single-blind, treatment purpose.	NSCLC	Concurrent	Adverse events, tumor lesion(s) on the MRI images, Measurement of BBB Opening.
NCT04021420	Safety and Efficacy of Blood Brain Barrier Opening With Implantable Device Sonocloud^®^ Combined With Nivolumab Used Alone or an Association With Ipilimumab in Brain Metastases From Patients With Malignant Melanoma	II	FUS + nivolumab	21	Single-group assignment; open-label; N/A allocation; treatment purpose.	Melanoma	Concurrent	Most Successful Dose (MSD), ORR, Intracranial overall response rate (ICORR), Extracranial overall response rate (BECORR)

## Data Availability

No datasets were generated or analysed during the current study.
